# Song Patterns and Stridulatory Movements in *Conocephalus* Species (Orthoptera: Tettigonioidea: Conocephalidae) of the Eastern Hemisphere: A Review with Some New Data

**DOI:** 10.3390/insects17101091

**Published:** 2026-10-21

**Authors:** Klaus-Gerhard Heller, Ahsan Ali, Ranjana Jaiswara

**Affiliations:** 1 Independent Researcher, 91732 Merkendorf, Germany; 2 Department of Zoology, Kargil Campus, University of Ladakh, Kargil 194105, India; ahsanalirahimi@gmail.com; 3 Department of Zoology, Panjab University, Chandigarh 160014, India; ranjana.jaiswara@gmail.com

**Keywords:** bioacoustics, song pattern, calling song, Conocephalidae, *Conocephalus maculatus*, *Conocephalus continuus*, *Conocephalus hilli* syn. nov

## Abstract

*Conocephalus* is a speciose, worldwide distributed genus of small bush-crickets (=katydids; Orthoptera, Tettigonioidea). While some of its species have huge ranges covering several continents, forms occurring in both hemispheres are not known. Partly due to their large ranges and because of morphological similarity, the correct identification of species is often not easy. Here, we present a review of the acoustic communication of all species with known songs in the eastern hemisphere. The species-specific amplitude pattern of the calling songs offers a helpful tool to find out which species are involved. Our results—using data from published sound files on compact discs, internet resources and conventional papers—show that species have the same species-specific songs all over their range. In addition to some misidentifications and situations which require further studies, however, we recover the widespread *Conocephalus maculatus* as a species complex with an Asiatic taxon (*C. maculatus* s.str.) and an African taxon (could be called *Conocephalus continuus*), both differing in song. The Indian *Conocephalus hilli* is considered a synonym (form) of *C. maculatus.*

## Simple Summary

To find a mate, in bush-crickets (=katydids) acoustic communication is essential. In the genus *Conocephalus*—and in many other bush-cricket species—the male sings, and the female localizes him by his species-specific song. These sounds can be used to identify species morphologically difficult to discriminate, but also to map their ranges. In this review, we present data on all *Conocephalus* species of the Eastern hemisphere with known song, based on recordings on published compact discs, on the internet and in conventional papers. Our results allow the acoustic identification of many species and confirm the findings that some species occur in more than one continent. However, one widespread and morphologically quite uniform form thought to live in Africa and Asia differs in song clearly between the continents and should now be called *C. maculatus* in Asia and *C. continuus* in Africa and Arabia. In some other species, unexpectedly large song differences have been observed, calling for more detailed studies.

## 1. Introduction

In the communication of many groups of Orthoptera, acoustic signals play a central role. This is especially true for bush-crickets (also called katydids; Tettigonioidea). Nearly all species of this group rely on sound signals for long-distance mate localization and attraction. Depending on the behavior and the ecology of the species involved, their signals—called calling songs—differ in many parameters. It is always the male who produces the calling song, but in Phaneropteridae, for example, the males often sing at a relatively low rate, and the females respond acoustically to the male song [1], whereas in Hetrodinae, the males produce long-lasting songs [2], which are used by the females to approach them phonotactically. This second type of communication system—singing males and phonotactically approaching females—is taxonomically much more widespread. Here, we review and describe the calling songs of the type genus of Conocephalidae, another group with mute females (often considered as subfamily Conocephalinae). Animals of this genus, *Conocephalus* Thunberg, 1815, are relatively small, slender tettigonioids (e.g., body length about 12–19 mm; [3]) and are grouped together with some similar forms into the tribe Conocephalini, which is currently considered monophyletic (but possibly not the genus *Conocephalus* itself; [4]).

The genus *Conocephalus* is found all around the world and includes more than 150 species [5]. According to present knowledge, it may have split off from the other Conocephalidae around 40 million years ago [6]. In our study, we present a review of the available acoustic data of all species of the genus; however, it is limited to the eastern hemisphere. All continents of this area are connected by common occurrences, whereas, according to our knowledge, at present, no species have been found native to both western and eastern hemispheres. In the eastern hemisphere, many species have large to very large ranges—e.g., *C. fuscus* from Europe to Japan, *C. maculatus* from Guinea to Japan, *C. laetus* from Mali to Australia and Vanuatu. These species often have long wings or at least forms with long wings (examples in [3]), so they may spread/be distributed relatively easily. On the other hand, many distribution maps look very patchy (see e.g., [5] for the three species listed above). For this, there are probably several reasons. The species may be rare and/or difficult to find, and there may be misidentifications in both directions—widespread species may not be recognized or may be identified as local species. In addition, identification from morphology is far from easy. In a worldwide study, out of 10 *Conocephalus* species studied genetically, only two could be identified morphologically to species level by the authors of the study [4]. Due to their large ranges, only very rarely large-scale studies were undertaken checking type specimens or at least material from distant localities (but see [7] for an outstanding positive exception). Since, within a single country, often, only a limited number of species is found, the interest in molecular studies seems also to be limited.

Studies of the species-specific calling songs may be a first step to get better data on which species occur where. This idea is supported by the observations that, within Europe, the song patterns of *C. fuscus* (as *C. discolor*) and *C. dorsalis* have been shown to be stable across the whole continent (e.g., [8,9]). In the present study, we will try to combine all available information about the amplitude modulation (=song pattern) of calling songs of *Conocephalus* in the eastern hemisphere. However, these data had to be collected from sources differing very widely in their intention. For example, they are presented as additions to more general studies like faunistic papers or on compact discs with the aim of giving an acoustic impression of the species involved. These limitations must be taken into account. For similar reasons, we will also not include data about the spectral composition of the song. It is well known that the songs of the small, delicate *Conocephalus* species contain strong ultrasonic components (e.g., [8,10]). The documentation of these frequencies requires special microphones not available in most studies, and even a high sampling rate is no proof of capturing the complete ultrasonic song spectrum. The presence of ultrasonic frequencies may affect the ratio of the amplitudes between opening and closing syllables, but this parameter does not seem to be diagnostic in *Conocephalus*.

## 2. Materials and Methods

For this review, all published song descriptions or digitally published songs of *Conocephalus* species of the eastern hemisphere that we had knowledge of were evaluated. Every source was considered as if the data were coming from one individual because of the mostly very limited information concerning the actual number of singing males studied. In addition, a few unpublished recordings were also included (Table 1; sound samples available in OSF under the respective species names).

The unpublished songs were recorded using a Racal store 4D tape recorder (London, UK) using Brüel and Kjær microphones 4133 and 4135 and amplifier 2606 ((Nærum, Denmark); frequency response flat up to 40 and 70 kHz respectively) or a Pettersson ultrasound detector D1000X (Uppsala, Sweden) distance microphone—animal 5–10 cm) or a Tascam Linear PCM Recorder (DR-05X MK II, TEAC Professional, Santa Fe Springs, CA, USA). Later, all recordings were digitized. The stridulatory movements were registered by an opto-electronic device ([11], modified as in [8]).

Song measurements were obtained using AUDACITY (http://audacity.sourceforge.net/), AMADEUS II and AMADEUS Pro (Martin Hairer; http://www.hairersoft.com) by analyzing the sound in the time-amplitude domain. In species with song descriptions, the same parameters (see below) were measured in the figures. Due to the above-mentioned limitations, no statistical evaluations were performed. For the figures, oscillograms of the songs were prepared using TURBOLAB (Bressner Technology, Puchheim, Germany).

### Bioacoustic Terminology (See Figure 1)

**Syllable** is the sound produced during one cycle of movements, generated by opening and closing of the tegmina, with each uni-directional movement generating a **hemisyllable**; **syllable duration** is the time period measured from the first impulse to the last; **syllable period** is the time period measured from the first impulse to the first impulse of the next syllable (reciprocal value: **syllable repetition rate [SRR]**); **impulse** is a simple, undivided transient train of sound waves (here the damped sound impulse arising as the effect of one tooth of the stridulatory file being struck by the scraper). A first-order assemblage of uniform syllables is called an **echeme** (=chirp; short series of syllables without stops of the wing movement in between), its length typically given in number of syllables; **echeme duration** is the time period measured from the first to the last syllable; **echeme period** is the time period measured from the first to the beginning of the next echeme (reciprocal value: **echeme repetition rate [ERR]**); a **trill** is a long series of uniform syllables without stops of the wing movement in between, its length typically given in seconds. However, there is no clear border between echemes and trills (broken trill, trill segments).

**Figure 1:**
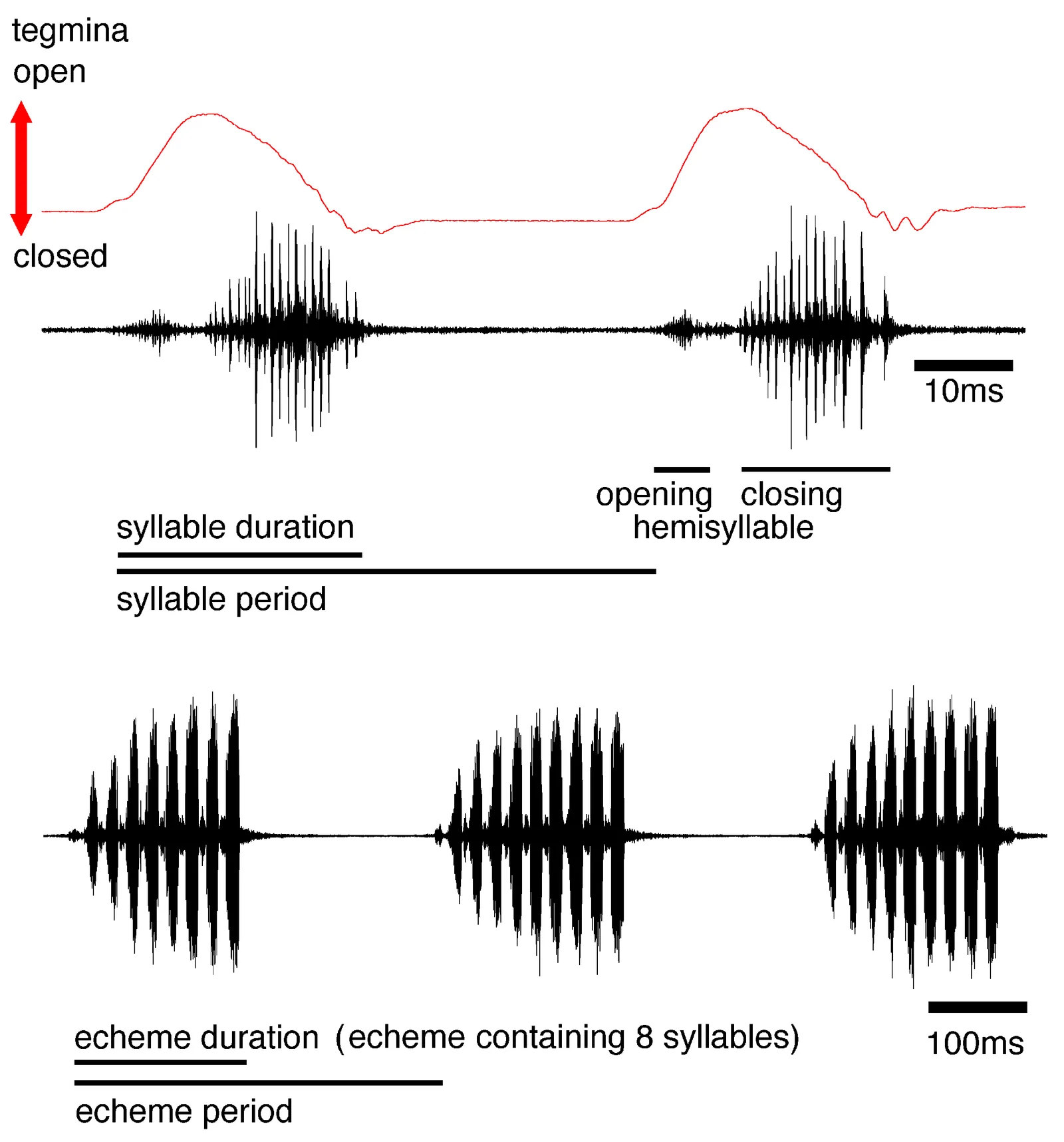
Schematic representation of the terms syllable ((**above**); stridulatory movement in red) and echeme (**below**) used for the song description (see Section 2; amplitude scale linear, in arbitrary units).

## 3. Results

For this review, we found data for 35 (sub-)species under the genus name *Conocephalus*. One of them was later transferred to *Euconocephalus*; for another (*C. adustus*), the data could not be obtained, and in a third recording from an unidentified species, no regular species-specific pattern could be recognized.

Among the remaining 32 forms, 21 produced echemes, six trills (in both cases half of the forms had simple, the other half complex songs), and another five produced a complicated mixture of trills and echemes. Below, the species are presented in alphabetical order according to the specific epithet. Subgenus names are provided only for information.

All species produced relatively long-lasting, continuous songs with a moderate to high duty cycle (syllable/echeme duration divided by syllable/echeme period). In most species, opening and closing movements of the forewings resulted in sound production, but always, the sounds from the closing movements had distinctly higher amplitudes. Only in *C. hastatus* and *C. kisi* could no opening hemisyllables be recognized.

*Conocephalus (Chloroxiphidion) albescens* (Walker, 1869)

Terra typica: South Australia. Distribution: Australia, New Zealand.

References to song: [12] (CD), this paper (Table 1).

**Figure 2:**
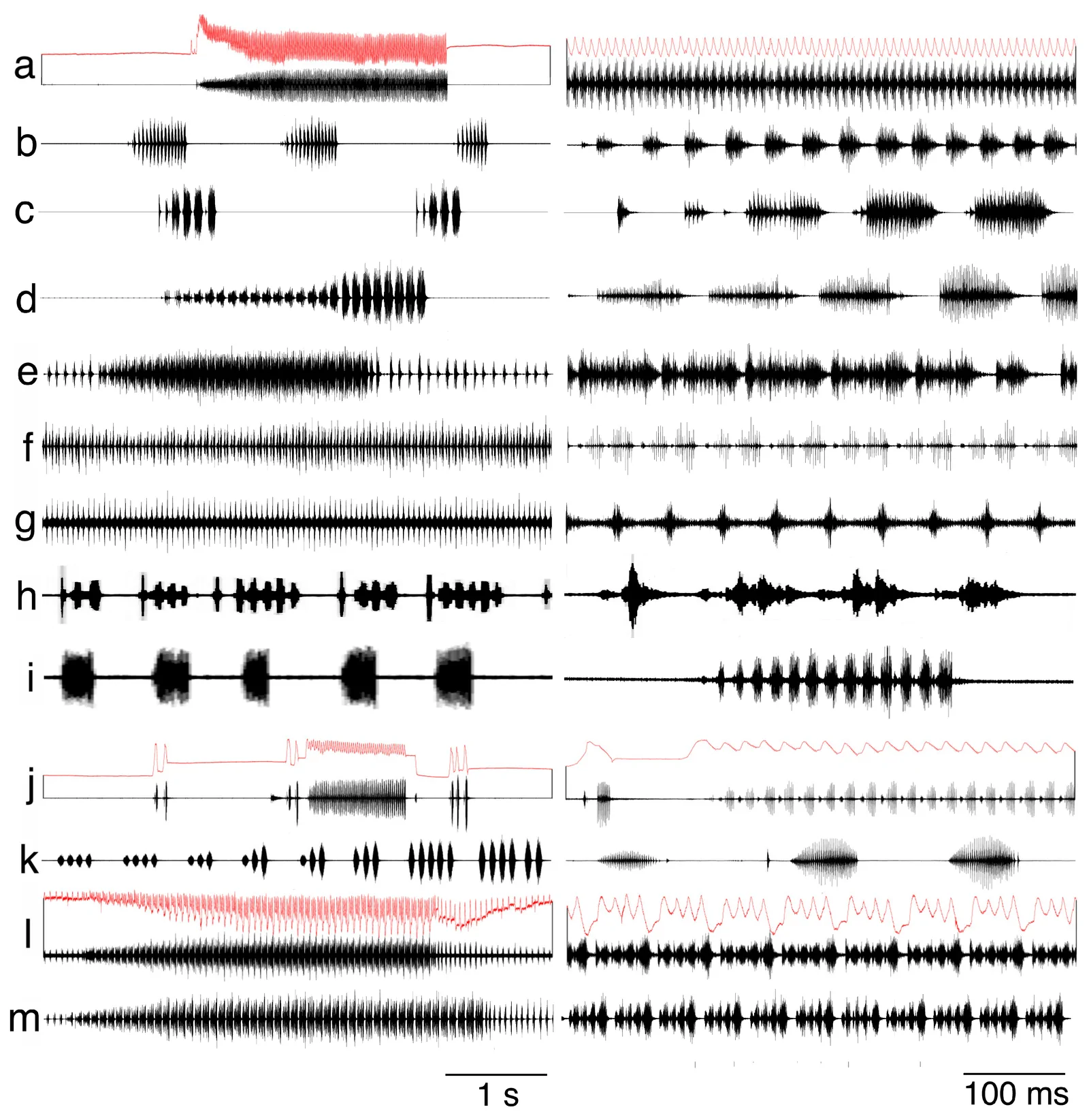
Oscillogram of calling song and tegmen movement (red; only in (**a**,**j**,**l**)) of *Conocephalus* species (left column 5-s-section, right column 500 ms section). (**a**)—*albescens*, (**b**)—*bambusanus* (Thailand; sound file [13] used under a Creative Commons Attribtion-ShareAlike 4.0 International License), (**c**)—*bambusanus* (Korea; sound file [15] in public domain *fide* Taewoo Kim), (**d**)—*bambusanus* (Japan; sound file [16] used with permission of Hideo Ueda), (**e**)—*beybienkoi* (sound file [16] used with permission of Hideo Ueda), (**f**)—*bilineatus* (sound file [12] used with permission of D. Rentz), (**g**)—*chinensis* (sound file [14] used with permission of Takashi Murai, Hokkaido University Press), (**h**)—*cognatus* (from [19], modified, with permission from the *Journal of Orthoptera Research*), (**i**)—*concolor (peneri)* (modified from Avci & Taylan (2023) [20], with permission from the *Journal of the Entomological Research Society*), (**j**)—*concolor hispanicus*, (**k**)—*conocephalus*, (**l**)—*dorsalis*, (**m**)—*ebneri* (sound file [28] used under a Creative Commons Attribtion-ShareAlike 4.0 International License) (see Table 2 for source of recordings).

The song consists of short (duration of a few seconds) trills with a relatively high SRR (67–111 Hz; Figure 2a; Table 2). The intervals between the trills have about the same length as the trills or are longer. The impulses produced during the opening movement of the tegmina are about 10 dB softer than those during the closing movements.

**Table 2:** Acoustical characteristics of the studied *Conocephalus* species (* source for figure or for locality data, respectively).

Species	Reference	SRR (Hz)	ERR (Hz)	*n* Syllables Per Echeme	T (°C)
*albescens*	this paper *	111	0.1		>24.5
*albescens*	[12]	67	0.3		20
*bambusanus*	[13] *	29	0.7	15	29
*bambusanus*	[14]	6	?	10	?
*bambusanus*	[15] *	8, 8	0, 4	5	?
*bambusanus*	[16] *	10	0.2	8, −30	?
*beybienkoi*	[16] *	61	13	4	?
*bilineatus*	[12] *	32	15.6	2	22
*chinensis*	[14] *	18.5	-	-	?
*chinensis*	[17]	20.8	-	-	25
*chinensis*	[15]	25	-	-	?
*chinensis*	[18]	19.2	-	-	26
*cognatus*	[19] *	40	8	2	30
*concolor (peneri)*	[20] *+email	55	1	14	?
*concolor hispanicus*	[8] *	50	see text	see text	>22
*conocephalus*	[21]	variable	see text		
*conocephalus*	[8]	variable	see text		
*conocephalus*	[9]	variable	see text		
*conocephalus*	[22]	variable	see text		
*conocephalus*	[23]	variable	see text		
*conocephalus*	[24]	variable	see text		
*conocephalus*	[25] *	11	see text	2–5	25
*conocephalus*	[26]	variable	see text		
*dorsalis*	[8] *	83	15.9	4	31
*ebneri*	[27]	80	16	4	22
*ebneri*	[28] *	67	13	4	28
*exemptus*	[29]	37	4.5	5	?
*exemptus*	[14,18] * (as *gladiatus*)	50	2.9	9	26
*exemptus*	[17]	60	4	8	28
*exemptus*	[15]	50	3, 3	8	?
*exemptus*	[19]	50	4	4–6?	29.4
*exemptus*	[16]	50	2.5	9	?
*fuscus*	[8], this paper *	50	12	3	>24
*fuscus*	[16]	35	8	3	?
*halophilus*	[16] *	38–55	see text	8–12	?
*hastatus hastatus*	[8] *	49	1	16	27
*hastatus hastatus*	[30]	45–56	0, 7–2	11	24–28
*iris*	[25] *	38	13	2	26
*iris*	[31]	28	12.7	2	?
*iris*	[32]	85	-	-	24
*japonicus*	[14]	48	4.5	6	?
*japonicus*	[15] *	48	4	5	?
*japonicus*	[16]	70	5	5	?
*kisi*	[8] *	55	0.7	25	>27
*laetus*	[12] *	22	0.5	10	29
*longipennis*	[33] *	70	1.5	ca. 25	25–29
*maculatus*	[34] *	77	11	4	31
*maculatus*	[14]	50	7.1	6	?
*maculatus*	[17]	54.3	5.3	10	29
*maculatus*	[15]	63	7.1	9	?
*maculatus*	this paper	56	5	8	30
*maculatus*	[35]	49	6	7–9	26.3
*maculatus*	[16]	60	5	10–30	?
*maculatus*	this paper (f. *hilli*) *	59	8.8	5	24
*(maculatus =>) continuus*	this paper	91	45	2	?
*(maculatus =>) continuus*	[36]	44	21	see text	?
*(maculatus =>) continuus*	[37]	56	28	2	?
*(maculatus =>) continuus*	[32]	85	see text	see text	26
*(maculatus =>) continuus*	[31] A *	80	38.5	2	>30
*(maculatus =>) continuus*	[31] B	43	22	2	>21.5
*melaenoides*	[38] *	73	28	2	25
*melaenus*	this paper	67	17.2	3	27
*melaenus*	this paper *	77	20.4	3	28
*melaenus*	[39]	67	19.6	3	28
*melaenus*	[38]	70	17.4	3	25
*melaenus*	[14]	71	17.5	3	26
*melaenus*	[17]	77.5	19	3	29
*melaenus*	[40]	67?	16	3	28.8
*melaenus*	[16]	60	12.5	3	?
*melaenus*	([16], as *chinensis*) *	67	15	3	?
*peringueyi*	[36] *	7	-	25	?
*phasma*	[31] *	36	1.45	10	20
*redtenbacheri*	[7]	118	-	see text	30
*redtenbacheri*	[12] *	67	-	see text	26
*semivittatus semivittatus*	this paper *	77	1	see text	>23.5
*semivittatus semivittatus*	[12]	45	0.45	see text	23
*semivittatus vittatus*	[41]	51	see text	see text	29
*semivittatus vittatus*	[7]	51	see text	see text	29
*semivittatus vittatus*	[42]	52	see text	see text	25–30
*signatus/trifasciatus*	[43] *	110	2, 6	15	28
*signatus/trifasciatus*	this paper *	60	8	3–6	28
*upoluensis*	[12] (A)	33	-	-	20
*upoluensis*	[12] (B)	62.5	-	-	27
*upoluensis*	[41]	81	-	-	21.5
*willemsei*	[12] *	17	0.83	10	23
sp.	[44]	11, 5–26	0, 5	5–7, many	24.6
*X (Conocephalus)*	[40]	174	-	-	?

*Conocephalus (Anisoptera) adustus* (Redtenbacher, 1891)

Terra typica: Indonesia, Ambon Island. Distribution: Indonesia, Maluku Islands, Ambon and Buru Islands.

References to song: [45]—New Record for Java Island.

Paper inaccessible (can only be read in the libraries of the Universitas Gadjah Mada at Yogyakarta or Jakarta; email 25 March 2026).

*Conocephalus (Conocephalus) bambusanus* Ingrisch, 1990

Terra typica: Thailand. Distribution: Southeast and Eastern Asia.

References to song: [13,14,15,16,46] (CD).

In Thailand, the song consists of echemes with up to 15 syllables (Figure 2b; Table 2). SRR varied relatively widely, between 17.8 and 37.0 Hz, with a mean of 26.8 Hz (syllable period: 37.3 ± 7.5 ms). The song of the animals from Korea (with quite low syllable numbers per echeme) and Japan, however, had a distinctly lower SRR of 6–9 Hz (Figure 2c). Sometimes (Figure 2d; see [16]), a male adds a series of additional syllables with a distinctly higher amplitude at the end of an echeme (see also *C. conocephalus*).

The difference in SRR is certainly too large to be explained by intraspecific variability, although the recording from Thailand was made at an unusually high temperature, and the temperatures of the other recordings are not given. Recordings at different temperatures have to confirm the assumption that two species are involved.

*C. bispinatus* Pitkin, 1980, and the later-described *C. bambusanus* share some important morphological characteristics that are rare or even unique in *Conocephalus*. Based on the high variability, Pitkin [7] excluded specimens from outside the Pacific region from the type series of *bispinatus*. Possibly, there are some more species in this group, besides *bambusanus*, not recognized because of the similarity in the key characters (see Section 4).

*Conocephalus (Anisoptera) beybienkoi* Storozhenko, 1981

Terra typica: Russian Far East. Distribution: Eastern Asia.

References to song: [16] (CD).

In the calling song, two different parts regularly alternate, with each lasting several to many seconds (Figure 2e; Table 2). One part consists of (tri- to ?) tetra-syllabic echemes (ERR 12.5 Hz, SRR 61 Hz). The other part consists of isolated syllables (SRR ca. 11 Hz). The song is thus quite similar to that of *C. dorsalis* (see below), supporting the close relationship of both forms. *C. beybienkoi* was originally described as a subspecies of *C. dorsalis* [47].

*Conocephalus (Anisoptera) bilineatus* (Erichson, 1842)

Terra typica: Australia. Distribution: Australia.

References to song: [12] (CD).

The song consists of a long sequence of di-syllabic echemes (SRR 32 Hz, ERR 15.6 Hz; Figure 2f; Table 2).

*Conocephalus (Amurocephalus) chinensis* (Redtenbacher, 1891)

Terra typica: Amur region. Distribution: Eastern Asia.

References to song: [14,15,16,17,18,46] (CD).

The song looks like it consists of trills (durations of at least between 4 and 60 s) with SRR of 18–25 Hz (Figure 2g; Table 2). However, the syllables are unusually short (7–8 ms; see [17]); thus, the tegmina may not move during the inter-syllable intervals and the song has to be classified as a very fast sequence of single syllables. In the recording of Kim [15], but not in the otherwise similar recording of Kim [46], the intervals between the syllables are filled with impulse-like sounds, which could suggest a more complicated movement pattern. The recording of Ueda [16] comes undoubtedly from a misidentified *C. melaenus* (see there).

*Conocephalus (Anisoptera) cognatus* (Redtenbacher, 1891)

Terra typica: Borneo & Ambon Island. Distribution: Southeast Asia.

References to song: [19].

The song of *C. cognatus* is unusually complicated (Figure 2h; Table 2). Each song unit starts with a loud isolated syllable which is followed—after about 100 ms—by a series of 2–5 di-syllabic echemes (SRR 40 Hz, ERR 8 Hz). In the isolated syllable opening and closing, hemisyllables are clearly recognizable, whereas in the echemes, it is more difficult. Possibly, the tegmina are not completely closed during this part. The song units are repeated in a rhythm of about 1 Hz.

*Conocephalus (Anisoptera) concolor* (Burmeister, 1838)

Terra typica: Egypt. Distribution: Mediterranean region and western Asia (several subspecies).

References to song: [20] (the song was described under the name *C. fuscus* but later corrected to *concolor*; email Taylan 30 August 2023). The recorded specimens may belong to the subspecies *Conocephalus concolor peneri* Harz, 1970 (see [48]).

The song consists of relatively long echemes (syllable number around 14, SRR 55 Hz), repeated about once per second (Figure 2i; Table 2).

*Conocephalus (Anisoptera) concolor hispanicus* Heller, 1988

Terra typica: Spain. Distribution: Spain.

References to song: [8].

The calling song is quite variable. It contains isolated syllables, often two closely together, and short trills (duration 0.1–1 s; SRR 50 Hz; Figure 2j; Table 2). At night, in the field, isolated syllables were more common; in the laboratory, both parts were heard at nearly the same frequency. During the trill, the amplitude of the stridulatory movement is typically clearly smaller than in the isolated syllables.

*Conocephalus (Conocephalus) conocephalus* (Linnaeus, 1767)

Terra typica: Africa. Distribution: Africa and southern Europe.

References to song: [8,9,21,22,23,24,25,26,27] (CD).

The song is very variable, from series of isolated syllables to echemes and even trills, and there are also differences between singing during daytime and at night (see [26,27]). Most typically, the song consists of long sequences of echemes with two to five syllables. As a characteristic feature, at the end of a sequence, the amplitude increases more or less abruptly (Figure 2k; Table 2) by around 10 dB. Compared to other *Conocephalus* species, the syllables are quite long, and SRR is low (often less than 10 Hz).

In the figure of Sales & Pye ([21]; pl. 10 a), an SRR of about 47 Hz can be recognized (see [8]). Most likely, here, an error in the identification of the species is involved, but there are no other recordings from these regions of Africa (Uganda, Nigeria) to clarify the situation.

*Conocephalus (Anisoptera) dorsalis* (Latreille, 1804)

Terra typica: France. Distribution: Palaearctic (introduced to North America).

References to song: [8,9,27] and many others from Europe (further references see in three listed studies).

In the calling song, two different parts alternate regularly, with each lasting several to many seconds. In both parts, the syllables contain distinct opening and closing hemisyllables. One part consists of tetrasyllabic echemes (ERR 11–17 Hz). In the first three syllables, the tegmina are not completely closed; only in the last do they come close to the resting position, resulting in a longer closing hemisyllable (SRR 70–80 Hz at 22 °C). Correspondingly, the next opening hemisyllable is longer and sometimes even louder than the following three (Figure 2l; Table 2). The other part consists of isolated syllables (SRR 15–30 Hz) with a decreasing amplitude of wing movement and sound.

*Conocephalus (Anisoptera) ebneri* Harz, 1966

Terra typica: Albania. Distribution: Montenegro, Albania, Greece.

References to song: [24,28].

The song is nearly identical to that of *Conocephalus dorsalis*—only rarely have a few echemes with five syllables been observed (Figure 2m; Table 2). In morphology, it looks like a very large *C. dorsalis* [3].

*Conocephalus (Anisoptera) exemptus* (Walker, 1869) (syn. *C. gladiatus* (Redtenbacher, 1891))

Terra typica: Korea. Distribution: Eastern Asia.

References to song: [14,15,16,17,19,29] (CD; as *gladiatus*).

**Figure 3:**
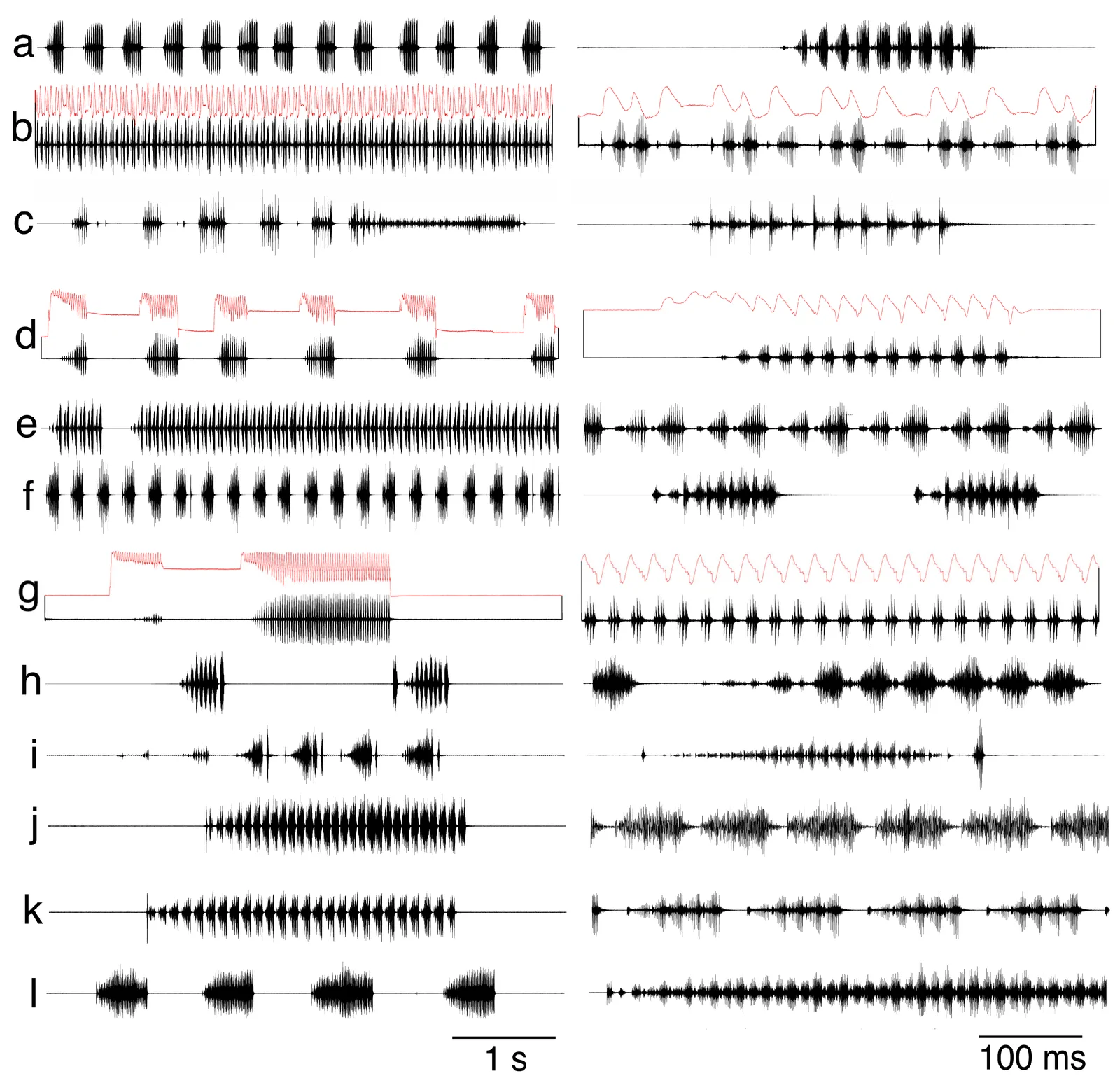
Oscillogram of calling song and tegmen movement (red; only in (**b**,**d**,**g**)) of *Conocephalus* species (left column 5-s-section, right column 500 ms section). (**a**)—*exemptus* (sound file [14] used with permission of Takashi Murai, Hokkaido University Press), (**b**)—*fuscus*, (**c**)—*halophilus* (sound file [16] used with permission of Hideo Ueda), (**d**)—*hastatus hastatus*, (**e**)—*iris*, (**f**)—*japonicus* (sound file [16] in public domain *fide* Taewoo Kim), (**g**)—*kisi*, (**h**)—*laetus* (sound file [12] used with permission of D. Rentz), (**i**)—*longipennis* (sound file [33] used under Common Creative CC zero license), (**j**)—*maculatus*, (**k**) *maculatus* (“*hilli*”), (**l**)—*continuus* (sound file [31] used with permission of E.Schweizerbart’sche Verlagsbuchhandlung: www.schweizerbart.de/9783510614189 (accessed on 5 April 2026)) (see Table 2 for source of recordings).

The song consists of echemes (ERR 3–4.5 Hz) containing five to nine syllables each (Figure 3a; Table 2). In the first half of an echeme, the syllables increase in amplitude, with the closing hemisyllables always being much louder than the opening (SRR 40–60 Hz). Tan et al. [19] report a trill-like sequence at the beginning of an echeme series, and this is also the only part of this recording where the SRR can be estimated to be around 50 Hz.

*Conocephalus (Anisoptera) fuscus* (Fabricius, 1793) (syn. *C. discolor* Thunberg, 1815)

Terra typica: Europe. Distribution: Palaearctis.

References to song: [8,9,27] (CD), this paper (Table 1) and many others from Europe (further references see in studies listed above), ref. [16] (CD).

The basic unit of the unique song pattern consists of the combination of a di-syllabic echeme (SRR ca. 50 Hz) and an isolated syllable. This element is repeated in a rhythm of about 10–16 Hz (Figure 3b; Table 2) for quite a long time (up to several minutes). Closing hemisyllables are distinctly louder and longer than opening hemisyllables. Many animals, but not all, insert short (0.3–2 s) series of isolated syllables into the long sequences, a behavior similar to *C. dorsalis*, but usually with shorter inserted syllable series.

*Conocephalus halophilus* Ishikawa, 2004

Terra typica: Japan. Distribution: Japan.

References to song: [16] (CD).

The basic unit of the song consists of a complex, amplitude-modulated combination of several echemes followed by a soft trill (Figure 3c; Table 2). In the first part, several (ca. 5) quite variable echemes are observed, separated by intervals of 300–500 ms. They contain 8–12 syllables with SRRs between 38–55 Hz. Then another echeme follows, during which the amplitude becomes softer and which ends in a long (1–1.5 s), soft trill, about 8–15 dB softer than the echeme. This combination is repeated after about 10 s. Sometimes, the echeme series and the trills are also presented separately.

*Conocephalus (Anisoptera) hastatus hastatus* (Charpentier, 1825)

Terra typica: Balkans. Distribution: Balkans, Asia Minor.

References to song: [8,24,30].

The song consists of short (0.3–1 s) trills (SRR ca. 50 Hz). Often, several of these syllable groups follow each other with intervals of less than 1 s, then a larger interval is observed. Sound is produced only during the closing movements, and the resting position of the tegmina between the trills can be variable (Figure 3d; Table 2).

*Conocephalus (Anisoptera) iris* (Serville, 1838)

Terra typica: Madagascar. Distribution: Sub-Saharan Africa, Madagascar.

References to song: [25,31,32].

“In the calling song of *C. iris* … two different song types can be observed. One consists of short echemes with 2–3 long syllables, which are presented in a slow syllable repetition rate (11–15 Hz…). The other type, which is much more common, consists of sequences of pairs of short syllables” (Figure 3e; Table 2). “The duration of these sequences is quite variable, from 0.1 to 4.2 s. The SRR within the pairs (38 Hz) is much higher than in the slow type, therefore it is called fast type; the repetition rate of the pairs in the fast type is comparable of that of the syllables in the slow type. The echemes of the slow type are either presented isolated with large intervals between or before series of syllable sequences of the fast type. These series can last many seconds, containing short and long sequences” [25]. This description is based on songs from Tanzania and West Africa [25,31], in Mozambique, the SRR seems to be much higher as calculated from the figure presented in Naskrecki & Guta [32].

*Conocephalus (Anisoptera) japonicus* (Redtenbacher, 1891)

Terra typica: Japan. Distribution: Eastern Asia.

References to song: [14,15,16,46] (CD).

The basic unit of the song is represented by an echeme containing five to six syllables (SRR 48 Hz). These echemes are repeated in small groups (three to six echemes; Japan: [14]) or in long uninterrupted series (Korea: [15]; Figure 3f; Table 2), always with an ERR of 4–4.5 Hz. Occasionally, single isolated syllables are interspersed. The first syllable of an echeme is often louder than the following, which increase in amplitude towards the end of the echeme. As can be seen from the structure of the syllables in the figures, during the opening and closing of the tegmina, impulses are produced.

*Conocephalus (Anisoptera) kisi* Harz, 1967

Terra typica: Balkans. Distribution: Balkans, Asia Minor.

References to song: [8,24] (same recording as 8).

The song consists of short (0.5–1 s) trills (SRR ca. 50 Hz), typically slightly longer than in *C. hastatus*. The sound is produced only during the closing movements, and the resting position of the tegmina between the trills can be variable (Figure 3g; Table 2). Sometimes, short and soft trills can be heard, resulting from wing movements with low amplitude. During acoustical rivalry between males, single syllables and short irregular syllable groups have been observed [8].

*Conocephalus (Chloroxiphidion) laetus* (Redtenbacher, 1891)

Terra typica: Australia. Distribution: West Africa to New Hebrides.

References to song: [12] (CD).

The song consists of echemes repeated at intervals of one to a few seconds. The echemes contain about 10 syllables (SRR ca. 23 Hz), but the first and last ones are a little bit separated and differ in structure (Figure 3h; Table 2). Pitkin [7] found the species morphologically very close to *C. exemptus* (listed by her under its synonym *C. gladiatus*), but the songs are quite different.

*Conocephalus (Anisoptera) longipennis* (de Haan, 1843)

Terra typica: Indonesia, Sumatra. Distribution: From Pakistan to Southeast Asia and Micronesia.

References to song: [33].

The basic unit of the song is a short trill, first increasing and then decreasing in amplitude, which is preceded by an isolated syllable and followed by a loud isolated syllable. SRR in the trill is ca. 70 Hz (*n* syllables ca. 25) and the isolated syllables are separated by intervals of ca. 15–20 ms (Figure 3i; Table 2). This basic unit is produced isolated or in short groups (up to six in a rhythm of about 1.5 Hz).

*Conocephalus (Anisoptera) maculatus* (Le Guillou, 1841)—complex

*C. maculatus* is a widespread species which can easily be recognized from coloration, and quite a number of recordings exist from different areas of its huge range. Surprisingly, however, two different song patterns are found, one in Asia and the other in Africa (compare Figure 3j–l). The differences are so large that obviously two sibling taxa are involved. The difference in the neuronal networks responsible for the production of the dissimilar songs can only be explained by the existence of separate gene pools, and the females will certainly not be attracted by the “wrong” song pattern. Therefore, two names should be used, *C. maculatus* for the Eastern forms and *Conocephalus continuus* (Walker, 1869) for the western forms, mainly from Africa. In her study on Pacific *Conocephalus*, Pitkin [7] had only two specimens of the *C. maculatus*-group from Africa, both with fastigia narrower than in all Asian specimens she measured, but she nevertheless synonymized the African *Conocephalus continuus* with the Asian *C. maculatus*.

*Conocephalus (Anisoptera) maculatus* (Le Guillou, 1841) [*s.str*.; see also below, *Conocephalus continuus* (Walker, 1869)]

- *Conocephalus (Anisoptera) hilli* Farooqi & Usmani, 2019 *syn. nov.*

Terra typica: West Malaysia (see [7]). Distribution: From Pakistan to Japan and Southeast Asia.

References to song: [14,15,16,18,21,34,35] (CD), this paper (Table 1).

The song consists of sequences of echemes (ERR 5–11 Hz), with each containing four to 10 syllables (SRR 49–77 Hz; Figure 3j,k; Table 2). The first syllable of a sequence is often louder than the following. The length of the sequences varies between 2 and 5 s.

*Conocephalus hilli* was described by Farooqi & Usmani [49] on three females, differing from *C. maculatus* mainly in length of the wings and length and shape of the ovipositor. However, wing length is a character that is well known for intraspecific variability in *Conocephalus* (see e.g., [3] for para-, macro- and hyperpterous specimens of *C. fuscus* and micro- and macropterous specimens in C. *dorsalis*). For ovipositor shape and length, no data are given, either by measurements or by figures, but according to the photos in Farooqi et al. [50], the differences from *maculatus* are quite small. Also, the differences in the number of stridulatory teeth, given in the description of the male ([50]: 40–42 in *hilli* to 50 in *maculatus*), are quite small. Since the songs of both forms are nearly identical (Figure 3j,k), and *C. hilli* is known only from one small area within the range of *maculatus* (see map in [50]), we consider *hilli* a local variety.

*Conocephalus (Anisoptera) continuus* (Walker, 1869) [see also above, *Conocephalus maculatus* (Le Guillou, 1841)]

Terra typica: Sierra Leone. Distribution: Africa to the United Arab Emirates.

References to song: [31,32,36,37] (all as *maculatus*), this paper (Table 1; see [51], Fontana per email 26 February 2026).

The song has a trill-like structure, but every second syllable is slightly softer and shorter than the preceding (in small figures sometimes not recognizable). Thus, it has to be classified as a sequence of di-syllabic echemes (ERR 22–44 Hz, SRR 44–100 Hz; when considered as a trill of 43–90 Hz; Figure 3l; Table 2). At present, the surprisingly large differences between some recordings have to be considered temperature effects (see Section 4). The length of the sequences varies between 0.5 and 2 s.

*Conocephalus (Anisoptera) melaenoides* Sänger & Helfert, 1995

Terra typica: Indonesia, Sulawesi. Distribution: Indonesia, Sulawesi.

References to song: [38].

**Figure 4:**
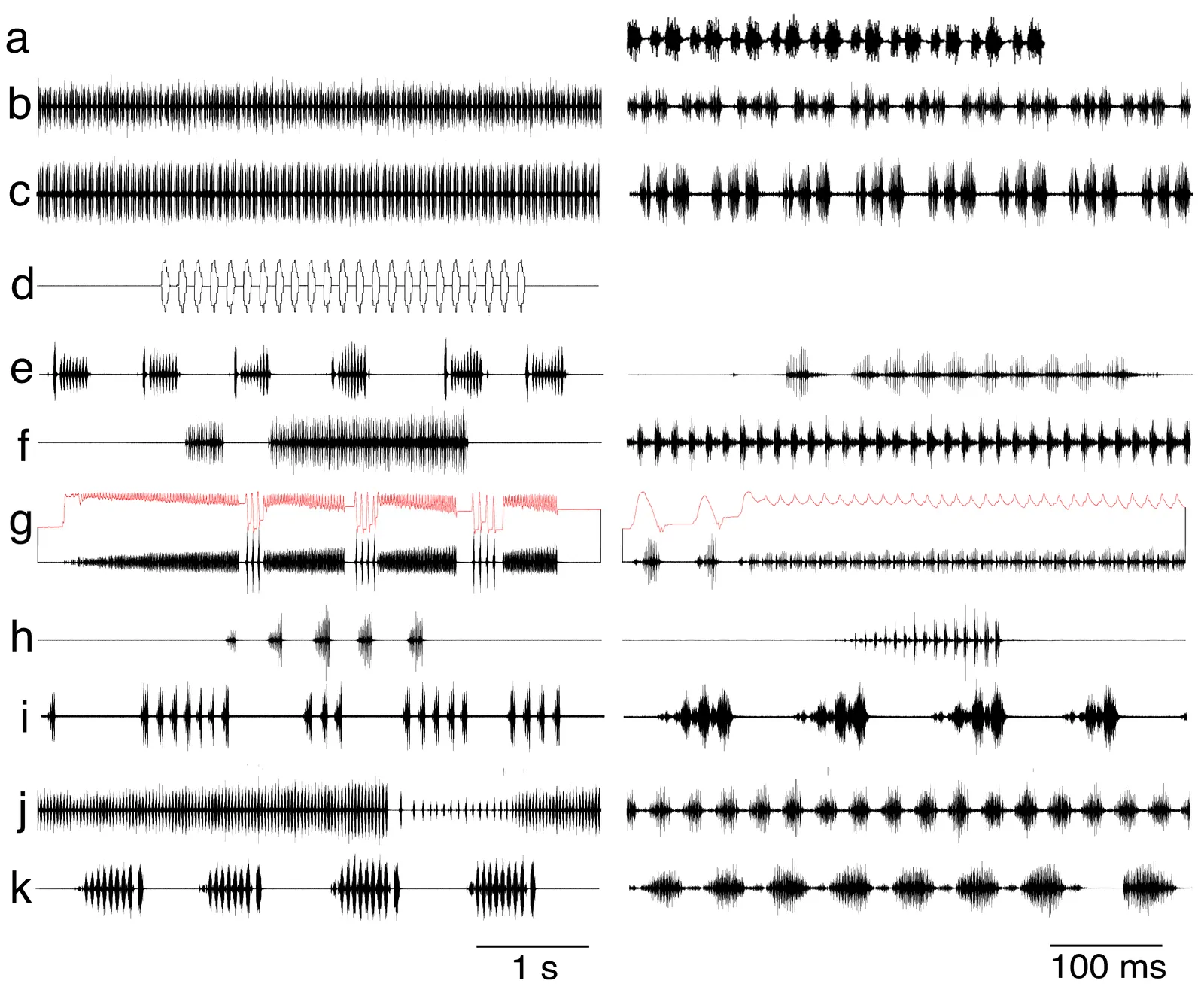
Oscillogram of calling song and tegmen movement (red; only in (**g**)) of *Conocephalus* species (left column 5-s-section, right column 500 ms section). (**a**)—*melaenoides* (from [38], modified, with permission from Zeitschrift der Arbeitsgemeinschaft österreichischer Entomologen and Brigitte Helfert), (**b**)—*melaenus*, (**c**)—*melaenus* (sound file [16] used with permission of Hideo Ueda), (**d**)—*peringueyi* (schematic oscillogramm based on data from [36]), (**e**)—*phasma* (sound file [31] used with permission of E.Schweizerbart’sche Verlagsbuchhandlung: www.schweizerbart.de/9783510614189, accessed on 5 April 2026), (**f**)—*redtenbacheri* (sound file [12] used with permission of D. Rentz), (**g**) *semivittatus semivittatus*, (**h**)—*signatus*/*trifasciatus* (sound file [43] used under Common Creative CC zero license), (**i**)—*signatus*/*trifasciatus* (this paper), (**j**)—*upoluensis*, (**k**)—*willemsei* (sound file [12] used with permission of D. Rentz) (see Table 2 for source of recordings).

The song consists of a sequence of di-syllabic echemes (ERR 28 Hz, SRR 73 Hz; Figure 4a; Table 2). In more detailed figures, the opening hemisyllables are visible, but they are much softer than the closing hemisyllables.

*Conocephalus (Anisoptera) melaenus* (Haan, 1843)

Terra typica: Indonesia, Java. Distribution: From India to China and Southeast Asia.

References to song: [14,16,17,18,38,39,40] (CD) this paper.

The song consists of long-lasting sequences (at least up to one minute) of strictly tri-syllabic echemes (ERR 15–20 Hz, SRR 65–80(–100?) Hz; Figure 4b,c; Table 2).

*Conocephalus (Anisoptera) peringueyi* Uvarov, 1928

Terra typica: South Africa. Distribution: South Africa.

References to song: [36,52].

As far as it can be concluded from the limited data, the song consists of echemes with variable duration and an unusually low SRR of about 7 Hz (Figure 4d; Table 2).

*Conocephalus (Megalotheca) phasma* Gorochov & Llorente del Moral, 2004

Terra typica: Tanzania. Distribution: Eastern Africa.

References to song: [31].

The basic unit of the song consists of a combination of an isolated syllable, followed after a very short interval (ca. 35 ms) by an echeme with ca. 10 syllables (SRR 36 Hz; Figure 4e; Table 2). The complete unit is often repeated in a rhythm of 1.5 Hz.

*Conocephalus (Anisoptera) redtenbacheri* (Bolívar, 1905)

Terra typica: New Guinea. Distribution: New Guinea to Australia, Micronesia and the Philippines.

References to song: [7,12] (CD).

The males produce short (up to 2–3 s) trills, sometimes irregular in length (Figure 4f; Table 2). SRR is quite high and can reach 118 Hz (at 30 °C, neotype recorded; only 67 Hz at 26 °C in Australia).

*Conocephalus (Anisoptera) semivittatus* (Walker, 1869)

Terra typica: New Zealand. Distribution: New Zealand to Australia, New Guinea, Micronesia and the Philippines.

References to song: *semivittatus semivittatus*: [12] (CD), this paper (Table 1); *s. semivittatus*?: [53]; *semivittatus vittatus*: [7,41,42].

The song consists of sequences (duration of about 5–20 s) during which trills and series of isolated syllables alternate (Figure 4g; Table 2). Typically, a sequence starts with a trill (duration often ca. 1 s; syllables slowly increasing in amplitude; SRR 50 Hz), which is followed by a short series of ca. three isolated syllables (SRR 20 Hz). These syllables have a much higher amplitude (by 6–8 dB) than the syllables in the trill. The combinations of trills and isolated syllables are repeated several times, with the number of isolated syllables often slightly increasing (e.g., from 3 to 6) and their SRR decreasing (e.g., to 12 Hz at the end). The impulses produced during the closing hemisyllables have a distinctly higher amplitude than those during the opening hemisyllables. This is true in the trill and in the isolated syllables. The high amplitude of the isolated syllables results from the fact that, here, the tegmina are closed to a larger degree than during the trill.

Bioacoustically, obvious differences between the subspecies *C. semivittatus semivittatus* and *C. semivittatus vittatus* (Redtenbacher, 1891) do not seem to exist.

*Conocephalus (Anisoptera) signatus* Redtenbacher 1891/*trifasciatus* Redtenbacher 1891

Terra typica: Sri Lanka. Distribution: Sri Lanka.

References to song: [43] (as possibly *trifasciatus*′), this paper (as cf. *trifasciatus*′; Table 1).

After recording, in both studies, the singing males were tentatively identified as belonging to the micropterous *C. trifasciatus*, probably from coloration. However, after studying long series, already, Henry [54] had concluded that it “seems more than probable that *X [iphidion] trifasciatus* will eventually fall as a synonym to *X. signatus*”. Henry [54] also lists the macropterous *C. maculatus* and *C. longipennis* from the island, but after this paper, obviously, no specimens of the genus *Conocephalus* have been examined in Sri Lanka.

In both recordings (both in the laboratory at 28 °C), the song consisted of echemes but with widely differing SRRs: Reynold’s male sang quite regular with 110 Hz (syllable number ca. 15, ERR 2.6 Hz; Figure 4h; Table 2) for more than 7 min, whereas, despite an observation period of several hours, our male rarely produced long series but mostly only isolated echemes with 60 Hz (syllable number 3–6, ERR up to 8 Hz, Figure 4i; Table 2; at 20 °C SRR 45 Hz, ERR typically of about 1 Hz or less). The differences between both recordings are certainly far outside intraspecific variability, and more detailed studies have to reveal the identity of the species described here.

*Conocephalus (Chloroxiphidion) upoluensis* (Karny, 1907)

Terra typica: Samoa. Distribution: Australia, Pacific Islands.

Reference to song: [12,42,55] (CD) (spectral data only).

Hugel [42] describes the song as follows: “The echeme-sequence of *C. upoluensis* call has two repeating distinct parts: a series of individual short syllables, alternated with a train of continuous buzz (this seems not to be always the case, see [12])”. Thus, in the song, two types of trills with different SRRs seem to alternate, both with durations of a few seconds. The faster (“buzz”) has an SRR of 81 Hz, the slower of 30.5 Hz (at 21.5 °C; [42]; Figure 4j, Table 2).

*Conocephalus (Anisoptera) willemsei* Pitkin, 1980

Terra typica: Australia, Northern Territory. Distribution: Australia.

Reference to song: [12] (CD).

The song consists of echemes (ERR 0.8 Hz) each containing about 10 syllables (SRR ca. 17 Hz). As a unique character, the last closing hemisyllable is separated from the others by a 30 ms long silent interval (Figure 4k, Table 2). This hemisyllable is also longer than the previous ones—probably, the amplitude of the tegmen movement is larger than before, and the wings return only then to their resting position.

*Conocephalus* sp.

Collecting locality: East Malaysia, Sabah.

Reference to song: [44].

In an 80 s recording, the male started with echemes (ERR 0.5 Hz; syllable number 5–7), which had at first an SRR of 26 Hz and which dropped quite fast to 12–13 Hz SRR. After about 40 s, the pattern changed to some trills (SRR 11–12; duration up to 3 s), until it ended in echemes again. Unfortunately, for the understanding of such a possibly complicated pattern, the sample size is too small.

*Conocephalus* X

Collecting locality: India: Assam, Hollangapar Gibbon Wildlife Sanctuary, Jorhat.

Reference to song: [40].

In the meantime, this species was described as *Euconocephalus brevicornis* Tiwari & Diwakar 2024. Its song, with a SRR of 174 Hz, also fits much better into *Euconocephalus* than into *Conocephalus*.

## 4. Discussion

Of the 140 species and subspecies of *Conocephalus* occurring in the eastern hemisphere [5], 32 could be studied here. They display a large variety of song patterns, but, as to be expected, in different recordings of the same species, an identical pattern is found—at least mostly. One of the most striking examples is *C. melaenus*, where, from India and Malaysia over to Thailand and China to Korea and Japan, everywhere, sequences of tri-syllabic echemes are found. For *C. exemptus*, the same seems to be true with a different pattern (4–9-syllabic echemes) in a perhaps somewhat smaller and more northern geographic range. In species with complex songs, the situation is similar; in *C. fuscus* (Europe to Japan), *C. dorsalis* (Europe to Franz Josef Land [5], Mongolia [56]) and *C. semivittatus* (New Zealand to Australia and New Guinea), all over their ranges, the same song can be heard. In the latter two, morphologically distinct subspecies are involved, whereas around *C. dorsalis,* even two closely related, allopatric species (*C. beybienkoi* and *C. ebneri*) share the same pattern. These two forms probably have to be considered subspecies of *dorsalis* under the “biological species concept” [57].

However, there are a few examples where species with the same name have different songs. The most surprising case concerns *C. maculatus*. From this extremely widespread species, quite a large number of recordings exist. They fall into two acoustically distinct and geographically separated groups. In Asia (India to Japan), all males produced sequences of four-to-10-syllabic echemes, whereas in Africa (and the United Arab Emirates) they make short trill-like sequences of di-syllabic echemes with their two syllables being so similar that they sometimes cannot be differentiated and look like a trill (see Figure 3j–l). On the basis of these structurally so different songs, we suggest that here, two species are involved. For the African “*C. maculatus*”, the name *C. continuus* (Walker 1869) could be used as the oldest available name. Both taxa represent a typical pair of ethospecies, known from many groups of animals and also from acoustically communicating Tettigonioidea [58].

There are two other cases with the same name for differently singing males. From Sri Lanka, two series of recordings exist, both ascribed as possibly or probably coming from *C. trilineatus* but differing very clearly in song structure (Figure 4h,i). This species differs only in coloration from the otherwise similar *C. signatus*. In addition, two similar species occur in the mainland of India just opposite Sri Lanka (*C. bivittatus* and *C. honorei* [59]; see [5]), making the situation even more complicated.

The last problematic example concerns *C. bambusanus*. This species is closely related to the New Guinean *C. bispinatus* Pitkin, 1980. Pitkin [7] herself had already material from Indochina and Borneo and considered the animals from both localities as possibly different from *C. bispinatus* but all “always having ventral spines on the mid femora” [7]. Ingrisch [60] described the animals from Thailand as new species and recorded also its song [13]. In 1992, Xia & Liu [61] described *C. abispinatus* from China, which was synonymized with *bambusanus* by Zhou et al. [62] and which also has the mid-femoral spines. Later, however, Kim & Kim [63] found specimens with some characteristics of *bambusanus* in Korea but mentioned that these animals did not have mid-femoral spines, the key character of *bambusanus* and *bispinatus*, but only an unarmed prosternum, as is typical for the whole subgenus *Conocephalus*. Nevertheless, they applied the name *bambusanus* to these animals. Storozhenko et al. [64], however, explain that all Korean specimens do have one or several mesofemoral spines. They even list these spines as typical for the subgenus, which is certainly not correct (see [7], own observations in *Conocephalus conocephalus*). From the Korean interpretation, the name may have come to Japan. Now, the songs of *bambusanus* in Thailand and in Korea and Japan are similar in structure but differ in rhythm (see Figure 2b,c). The East Asian recordings are much slower than those from Thailand. Such “speed” differences, however, can be caused by temperature differences at the time of recording (see [65]). Unfortunately, the temperatures during recording are not known for any of the East Asian recordings, whereas that from Thailand was unusually high. Judging from the size of the differences, it seems likely that different species are involved, but more detailed studies are necessary.

Songs of *Conocephalus* species are especially susceptible to temperature effects. Many *Conocephalus* species are active and singing during daytime when they are exposed to direct sunlight. In this situation, the animals may warm up and become hotter than the surrounding air. The increase in temperature depends on the duration of the exposure and the intensity of the sunlight, but it cannot easily be quantified or calculated by measuring the temperature of the ambient air. Thermal radiation thermometers may reduce the problem, but they have not been available in former times. However, also, the opposite effect may occur. *Conocephalus* species often occur in relatively moist environments. While sitting in moist vegetation, their body temperature will go down by evaporative cooling. This effect was observed by Bauer (pers. communication) during her experiments working with thermocouples in acridid grasshoppers [66]. Therefore, even songs, recorded at the same ambient temperature, may show considerable differences. These effects, for example, may possibly be responsible for the observed song differences in *C. iris* and *C. upoluensis*.

The species-specific patterns of male calling songs, from relatively simple to complicated, have to be received, perceived and evaluated by the females—and possibly by rival males. At present, it is unknown which characters of the song exactly are used by the animals for the recognition of conspecifics. In close-range acoustical tests, *Conocephalus* females always preferred the species-specific SRRs [67]. They do not seem to be able to resolve faster song structures like impulse intervals [68]. For one species (*C. nigropleurum* (Bruner, 1891)), a null-reaction to the pattern of a congeneric species is described, even when the song contained generally attractive frequencies [69]. On the other hand, *Conocephalus* has never been tested whether, for species recognition, the animals may concatenate, e.g., pairs of syllables, as is known for the bush-cricket *Tettigonia viridissima* (Linnaeus, 1758) [70]. For the complicated songs, we are very far from an understanding of the function of single elements.

Similarly, we do not yet have much information about taxonomic relationships and song patterns. Pairs (or groups) of related, allopatric species may share the same pattern, as seen in *dorsalis, beybienkoi* and *ebneri*. Interestingly, the two studied members of the subgenus *Conocephalus*, *conocephalus* and *bambusanus*, both have low SRRs and regular changes in amplitude, which could indicate a common origin. However, regular changes in loudness are possibly found only in the East Asian populations of *bambusanus*, and low SRRs are also observed in other species, such as *C. peringueyi*. Generally, information about the phylogeny of *Conocephalus* is completely missing except for the data in Mugleston et al. [4], which indicate mainly intracontinental relationships (Africa, America, Asia/Australia; eight of 10 species unidentified at the species level). Thus, in many fields of research (e.g., behavioral studies, physiology, molecular genetics, systematics), much has to be done to get a better understanding of the details of the function and the evolution of the calling songs in *Conocephalus*.

## Figures and Tables

**Table 1:** Locality data of the previously unpublished sound data and specimens (# specimen number in collection Heller).

Species	Locality	Recordist	Specimen #
*albescens* (Walker, 1869)	AUSTRALIA: New South Wales, Sydney (33°51′ S, 151°12′ E), 1–31 xii 1982, coll. F. Geiser	Heller, K.-G.	CH4169
*fuscus* (Fabricius, 1793)	GREECE: Preveza, Nikopolis (39°2′ N, 20°49′ E), 10 m, 1 vii 1986, coll. Heller	Heller, K.-G.	CH1276
*maculatus* (Le Guillou, 1841)	SRI LANKA: Anuradhapura (8°21′ N, 80°23′ E), 5 m, 3 iii 2014, recorded K.-G. Heller	Heller, K.-G.	CHX362
*maculatus* (Le Guillou, 1841) (“*hilli*”)	INDIA: Ladakh, Kargil, Chutumail Doks, Yarkian Mulook (34°33′12″ N; 76°10′34″ E), 2912 m a.s.l., 11.vi.2021, coll. Ahsan Ali	Jaiswara, R.	
*continuus* (Walker, 1869)	UNITED ARAB EMIRATES: Wadi Madaq, 3 vii 2007, coll. P. Fontana	Fontana P.	
*melaenus* (Haan, 1843)	MALAYSIA: Selangor, Ulu Gombak Field Study Centre (20 km nno Kuala Lumpur) (3°20′ N, 101°45′ E), 260 m, 8–28 iii 1981, coll. K.-G. Heller & M. Volleth	Heller, K.-G.	CH3719
*melaenus* (Haan, 1843)	MALAYSIA: Pahang, Kuala Lompat (westl. Kuala Kerau; Krau Wildlife Reserve) (3°43′ N, 102°16′ E), 18–21 iii 1992, coll. K.-G. Heller & M. Volleth	Heller, K.-G.	CH3401
*semivittatus semivittatus* (Walker, 1869)	AUSTRALIA: New South Wales, Sydney (33°51′ S, 151°12′ E), 1–31 xii 1982, coll. F. Geiser	Heller, K.-G.	CH4170
*signatus/trifasciatus*	SRI LANKA: Ella (8°52′ N, 81°3′ E), 500 m, 8 iii 2014, coll. M. + K.-G. Heller	Heller, K.-G.	CH7721

## Data Availability

The unpublished original data presented in the study are openly available in OrthopteraSpeciesFile (OSF) at http://orthoptera.speciesfile.org/.

## Abbreviations

The following abbreviations are used in this manuscript:

ERR	Echeme repetition rate
SRR	Syllable repetition rate
CD	Compact disc
